# PAH- and PCB-induced Alterations of Protein Tyrosine Kinase and Cytokine Gene Transcription in Harbor Seal (*Phoca Vitulina*) PBMC

**DOI:** 10.1080/17402520500116624

**Published:** 2005-06

**Authors:** Jennifer C. C. Neale, Thomas P. Kenny, Ronald S. Tjeerdema, M. Eric Gershwin

**Affiliations:** 1 Division of Rheumatology Allergy, and Clinical Immunology University of California One Shields Avenue Davis CA 95616 USA; 2 Department of Environmental Toxicology University of California One Shields Avenue Davis CA 95616 USA

## Abstract

Mechanisms underlying *in vitro* immunomodulatory effects of polycyclic aromatic hydrocarbons (PAHs) and polychlorinated biphenyls (PCBs) were investigated in harbor seal peripheral leukocytes, via real-time PCR. We examined the relative genetic expression of the protein tyrosine kinases (PTKs) *Fyn* and *Itk*, which play a critical role in T cell activation, and IL-2, a cytokine of central importance in initiating adaptive immune responses. IL-1, the macrophage-derived pro-inflammatory cytokine of innate immunity, was also included as a measure of macrophage function. Harbor seal PBMC were exposed to the prototypic immunotoxic PAH benzo[a]pyrene (BaP), 3,3',4,4',5,5'-hexachlorobiphenyl (CB-169), a model immunotoxic PCB, or DMSO (vehicle control). Exposure of Con A-stimulated harbor seal PBMC to both BaP and CB-169 produced significantly altered expression in all four targets relative to vehicle controls. The PTKs *Fyn* and *Itk* were both up-regulated following exposure to BaP and CB-169. In contrast, transcripts for IL-2 and IL-1 were decreased relative to controls by both treatments. Our findings are consistent with those of previous researchers working with human and rodent systems and support a hypothesis of contaminant-altered lymphocyte function mediated (at least in part) by disruption of T cell receptor (TCR) signaling and cytokine production.


*Clinical & Developmental Immunology*, June 2005; 12(2): 91–97

Taylor & Francis

Taylor & Francis Group

### PAH- and PCB-induced alterations of protein tyrosine kinase and cytokine gene transcription in harbor seal (*Phoca vitulina*) PBMC

JENNIFER C.C. NEALE[1], THOMAS P. KENNY[1], RONALD S. TJEERDEMA[2], & M. ERIC GERSHWIN[1]

[1]*Division of Rheumatology, Allergy, and Clinical Immunology, University of California, One Shields Avenue, Davis, CA, 95616, USA, and* [2]*Department of Environmental Toxicology, University of California, One Shields Avenue, Davis, CA, 95616, USA*

**Abstract**
Mechanisms underlying *in vitro* immunomodulatory effects of polycyclic aromatic hydrocarbons (PAHs) and polychlorinated biphenyls (PCBs) were investigated in harbor seal peripheral leukocytes, via real-time PCR. We examined the relative genetic expression of the protein tyrosine kinases (PTKs) *Fyn* and *Itk*, which play a critical role in T cell activation, and IL-2, a cytokine of central importance in initiating adaptive immune responses. IL-1, the macrophage-derived pro-inflammatory cytokine of innate immunity, was also included as a measure of macrophage function. Harbor seal PBMC were exposed to the prototypic immunotoxic PAH benzo[*a*]pyrene (BaP), 3,3′,4,4′,5,5′-hexachlorobiphenyl (CB-169), a model immunotoxic PCB, or DMSO (vehicle control). Exposure of Con A-stimulated harbor seal PBMC to both BaP and CB-169 produced significantly altered expression in all four targets relative to vehicle controls. The PTKs *Fyn* and *Itk* were both up-regulated following exposure to BaP and CB-169. In contrast, transcripts for IL-2 and IL-1 were decreased relative to controls by both treatments. Our findings are consistent with those of previous researchers working with human and rodent systems and support a hypothesis of contaminant-altered lymphocyte function mediated (at least in part) by disruption of T cell receptor (TCR) signaling and cytokine production.

**Keywords:** *Polycyclic aromatic hydrocarbon, polychlorinated biphenyl, Phoca vitulina, protein tyrosine kinase, cytokine, interleukin*

### Introduction

The immunotoxic effects of polycyclic aromatic hydrocarbons (PAHs) and halogenated aromatic hydrocarbons (HAHs), such as polychlorinated biphenyls (PCBs), have been investigated in rodent and human systems where they have been found to induce a broad range of adverse effects (reviewed in White 1986, Vos and Luster 1989, Kerkvliet and Burleson 1994, White et al. 1994). These ubiquitous marine pollutants tend to bioaccumulate through the food web and concentrate in tissues of marine mammals, whose long life span and high content of adipose tissue make them an ultimate sink for lipophilic contaminants. The highest levels are found in species, such as the harbor seal (*Phoca vitulina*), occupying top trophic levels and inhabiting heavily polluted coastal waters. PAHs and HAHs have been demonstrated to decrease host resistance to bacterial, viral, and parasitic disease in rodent models, and contaminant-induced immunosuppression is speculated to have contributed to the high mortality observed in several marine mammal populations during recent morbillivirus epizootics (Osterhaus and Vedder 1989, Hall et al. 1992, Aguilar and Borrell 1994, Tanabe et al. 1994, Van Loveren et al. 2000).

Altered immune parameters associated with environmental exposure to PAHs and PCBs have been reported for several species of pinnipeds and cetaceans and include reduced antigen-specific antibody responses, depressed T-lymphocyte proliferative

Correspondence: J. C. C. Neale, Division of Rheumatology, Allergy, and Clinical Immunology, University of California, One Shields Avenue, Davis, CA, 95616, USA. Tel: 1 530 752 3286. E-mail: jcneale@ucdavis.edu

ISSN 1740-2522 print/ISSN 1740-2530 online © 2005 Taylor & Francis Group Ltd
DOI: 10.1080/17402520500116624

responses to mitogen, depressed plasma retinol, increased white blood cell count and depressed red cell parameters, and frequent infections with mildly pathogenic bacteria and high rates of cancer (Martineau et al. 1994, Lahvis et al. 1995, Beckmen et al. 2003, Jenssen et al. 2003, Neale et al. 2005). Experiments in which captive harbor seals were fed heavily-contaminated (especially with PCBs) fish also supported a hypothesis of contaminant-induced immunological impairment. In addition to an increase in neutrophil counts in peripheral blood, the following parameters were depressed in seals fed contaminated fish: plasma retinol and thyroid hormone levels, natural killer cell activity, mitogen- and antigen-induced T cell proliferative responses, mixed lymphocyte reaction, and delayed-type hypersensitivity and specific serum antibody responses (Reijnders 1986, Brouwer et al. 1989, De Swart et al. 1996, Ross et al. 1996, Van Loveren et al. 2000).

Specific mechanisms of PAH- and PCB-induced immunotoxicity in these organisms, however, remain largely unexplored. Signal transduction and the T lymphocyte/IL-2 pathway have been proposed as sensitive targets of both PAHs and HAHs (Exon et al. 1985, House et al. 1987, Kerkvliet and Brauner 1987, House et al. 1989, Clark et al. 1991, Davila et al. 1995). The removal of pathogens is largely dependent on the process of T cell activation, proliferation, and differentiation into armed effector and memory cells. Activation of T lymphocytes via T cell receptor (TCR) signaling begins with protein tyrosine kinase (PTK) activation, which initiates a cascade of intracellular signaling that transfers the signal to other molecules and eventually carries it to the nucleus, where gene transcription for the production of cytokines and other mediators of lymphocyte function occurs (a very similar process occurs in B cells). Previously, we demonstrated that certain PCB and PAH compounds suppressed in vitro mitogen-stimulated T cell proliferation in the harbor seal (Neale et al. 2002). To explore potential mechanisms of this effect, here we exposed harbor seal leukocytes to the prototypic immunotoxic PAH, benzo[*a*]pyrene (BaP), and 3,3′,4,4′,5,5′-hexachlorobiphenyl (CB-169), a model immunotoxic PCB, in vitro, and examined, via real-time PCR, the relative expression of certain genes important in T cell activation and function.

We selected targets relevant to early TCR signaling and downstream cytokine production (these topics are reviewed in Weiss and Litmann 1994, Kung and Thomas 1997, Qian and Weiss 1997, Janeway et al. 1999, Abbas and Lichtman 2003). These included the PTKs *Fyn* and *Itk*, and the T cell growth factor, IL-2. The earliest biochemical events within the T cell that follow clustering of the TCR complex and coreceptors upon antigen stimulation are (1) the activation of PTKs including the Src-family kinases *Fyn* and *Lck*, associated with the cytoplasmic domains of the clustered CD3 and coreceptor proteins, and (2) phosphorylation by these PTKs of tyrosines in the ITAMs of the CD3 and ζ chains. ZAP-70 binds to phosphotyrosines of the ζ chain and phosphorylates adapter proteins. The Tec family kinase *Itk*, once activated, binds phosphorylated adapter proteins and can then phosphorylate and activate phospholipase C gamma 1 (PLCγ1). Active PLCγ1, in turn, hydrolyzes membrane $PIP_2$ to generate $IP_3$ and DAG, which then activate two distinct downstream signaling pathways in T cells. A third signaling pathway following phosphorylation of the adapter protein LAT by ZAP-70 is the Ras-MAP kinase pathway. All three eventually converge to generate active transcription factors that stimulate expression of various genes responsible for cellular responses.

Among the earliest detectable cellular responses to antigen recognition and TCR signaling is the secretion of cytokines. IL-2, the growth factor for antigen-stimulated T cells, is responsible for T cell clonal expansion after antigen recognition, primarily through autocrine activity. IL-2 also increases synthesis of other cytokines in T cells, promotes the proliferation and differentiation of NK cells, and acts as a growth factor and stimulus for antibody synthesis in B cells. In addition, we investigated genetic expression of IL-1, the macrophage-derived pro-inflammatory cytokine of innate immunity, as a measure of macrophage function. We included this target cytokine not only because the macrophage has been suggested as a primary target of PAH immunotoxicity in previous studies (Myers et al. 1987, 1988), but also because of the numerous roles served by macrophages in the effector phases of adaptive immune responses.

### Materials and methods

Free-ranging harbor seals (2 female pups, 1 male pup, 1 female yearling) were captured using tangle nets near a haul-out site in San Francisco Bay, California, on 5/31/04 and 6/1/04 (NMFS Scientific Research Permit No. 555-1565). Seals were physically restrained. Blood was drawn from the extradural venous sinus into sterile evacuated blood collection tubes containing acid citrate dextrose. Peripheral blood mononuclear cells (PBMC) were isolated from whole blood within 8 h of collection following methods detailed previously (Neale et al. 2002, 2004). White cell pellets were suspended in complete medium (RPMI 1640 medium with 2 mM L-glutamine, 0.08% gentamicin, and 10% fetal bovine serum). Cell viability was assessed via trypan blue exclusion and viable cells counted microscopically using a hemacytometer. PBMC ($4 \times 10^6$ cells/ml) were incubated (37°C, 5% $CO_2$, humidified atmosphere) in 48-well microtiter plates containing 250 μl cell suspension per well.

Following overnight incubation, wells were supplemented with 250 µl medium solutions containing concanavalin A (Con A; Sigma-Aldrich, St. Louis, MO, USA), a polyclonal T cell activator in the harbor seal, and either BaP (Sigma-Aldrich), CB-169 (AccuStandard, New Haven, CT, USA), or the vehicle control (anhydrous dimethylsulfoxide, DMSO; Sigma-Aldrich), in triplicate for each individual and treatment. Final concentrations were 5 µg/ml Con A and 20 µM BaP, CB-169, or DMSO. Cells were incubated for 4 h. Plates were then centrifuged at 200*g* for 2 min, supernatants were removed, and 250 µl TRIzol reagent (GibcoBrl/Life Technologies, Gaithersburg, MD, USA) added to each well with repetitive pipetting to ensure complete cell lysis. After 5 min incubation at room temperature, contents of wells were transferred to 1.5-ml microcentrifuge tubes. RNA was isolated (phenol/chloroform separation) and precipitated from the aqueous phase with isopropanol, according to the manufacturer's instructions. The RNA pellet (approximately 20 µg) was then redissolved in 20 µl diethyl-pyrocarbonate (DEPC)-treated RNase-free water (hereafter, "water"), pooled from triplicate samples, and incubated at 60°C for 10 min.

For reverse transcription, approximately 5 µg RNA (in 5 µl water) was added to a mixture of 1 µl (0.5 µg) oligo(dT)$_{12-18}$ primers, 1 µl dNTP Mix (10 mM), and 5 µl water. This mixture was incubated at 70°C for 10 min then cooled on ice. Next, 4 µl of 5 × First Strand Buffer, 2 µl of DTT (0.1 M) and 1 µl (10 units) RNase inhibitor were added to the first mixture and incubated at 42°C for 2 min. Finally, 1 µl (200 units) Superscript II reverse transcriptase was added for a total volume of 20 µl. This was incubated for 42°C for 50 min. The reverse transcriptase enzyme was heat-killed at 70°C for 15 min. All RT reagents supplied from Invitrogen (Carlsbad, CA, USA).

We designed the following real-time PCR primers (5′–3′) for amplification of harbor seal cDNA (forward/reverse): IL-1β, TGACCT GTACCCAGAGAGTCCA/AAACCCTTCTATTCCCCTTCCA; IL-2, GAAACTAAAGGGATCTGAAACA/GTGT TGAGAAGATGCTTTGAC; *Fyn*, CTTCGGATTGGCCAGATTGA/CCTTGCCTTGCTGTGTACTCG; *Itk*, GGGATGACAAGGTTCGTCCTT/GGTGCCTGTGGAACTGGTGT; and the internal control/housekeeping gene, beta-2-microglobulin (B2M), TGCTATGTGTCTGGGTTCCA/AAGGTAGAAAGACCAGTCCTTG. Primers were synthesized by Proligo (IL-2, FYN, ITK, B2M) and Invitrogen (IL-1). Primers for harbor seal IL-1β, *Fyn* and *Itk* were based on published harbor seal mRNA sequences [GenBank acc. AY578791, Bozza and Atkinson, unpublished (IL-1); GenBank acc. #AY611616 (*Fyn*) and #AY611617 (*Itk*), Neale et al. 2004]. IL-2 primers were derived from the nucleotide sequence from another phocid, the northern elephant seal (*Mirounga angustirostris*; GenBank acc. # U79187; Shoda et al. 1998). Carnivore sequences were not available for B2M, therefore these primers were derived from multiple consensus sequences from primate, pig, sheep, cow, rat and mouse.

PCR reactions (25 µl) were assembled in 96-well optical reaction plates with each well containing 1 µl cDNA (diluted 1:5 for *Fyn* and *Itk*, 1:10 for IL-1, IL-2), 1.5 µl each of 5 µM forward and reverse primers, 8.5 µl water, and 12.5 µl iTaq SYBR Green Supermix with ROX internal reference dye (Bio-Rad Laboratories, Hercules, CA, USA). Wells were covered with optical caps and plates were centrifuged for 3 min at 100*g* before loading into instrument. Real-time PCR was conducted and data analyzed with the 7900 ABI Prism Sequence Detection System and SDS 2.1 software (Applied Biosystems, Foster City, CA, USA). Cycling conditions (95°C, 3 min; 95°C 15 s, 60°C [57°C for IL-2] 1 min, 40 cycles) included a melt curve analysis (95°C 15 s, 60°C 15 s). Duplicate samples and triplicate no-template controls were included for each set of primers. A validation experiment was performed using serial dilutions of untreated cDNA from one individual to confirm equivalent relative efficiencies for target and housekeeping genes. Amplicon identities were supported via agarose gel electrophoresis of PCR products which demonstrated single bands approximating the expected sizes (IL-1, 71 bp; IL-2, 113 bp; B2M, 120 bp; *Fyn* and *Itk*, 51 bp).

Replicate raw data (threshold cycle number, or $C_t$) for each sample were averaged and then adjusted by dividing these values by the corresponding averaged $C_t$ for B2M to correct for any differences in starting quantity of material. These ratios were then compared between treatments and controls using 2-tailed, paired (i.e. within seal) *t*-tests. To strengthen our analysis, we performed identical real-time PCR using another common housekeeping gene, glyceraldehyde-3-phosphate dehydrogenase, for target gene adjustment; those results were qualitatively and quantitatively similar to those obtained using B2M correction and are therefore not presented here.

### Results

Expression of PTKs was less variable among individuals than expression of interleukins (Table I). *Fyn* was least variable and IL-2 most variable overall; greatest inter-individual differences were observed in BaP and DMSO exposures for IL-2, related largely to lower Ct ratios (corresponding to greater cDNA copy number) for Harbor Seal 2. PBMCs from this individual also produced greater IL-1 transcripts in all samples than those of the other three seals.

Exposure of Con A-stimulated harbor seal PBMC to both the model PAH and PCB produced significantly altered expression in all four targets relative to vehicle controls (Figure 1). *Fyn* and *Itk* were

Table I. Gene expression and standard deviation of protein tyrosine kinases *Fyn* and *Itk* and cytokines IL-2 and IL-1 in Con A-stimulated harbor seal PBMC exposed *in vitro* to 20 μM benzo[*a*]pyrene (BaP), 20 μM hexachlorobiphenyl 169, or vehicle control (20 μM DMSO), based on real-time PCR.

| | *Fyn* | | | *Itk* | | | IL-2 | | | IL-1 | | |
|---|---|---|---|---|---|---|---|---|---|---|---|---|
| | BaP | CB-169 | Control | BaP | CB-169 | Control | BaP | CB-169 | Control | BaP | CB-169 | Control |
| HS1 | 1.16 | 1.12 | 1.17 | 1.20 | 1.23 | 1.32 | 1.38 | 1.07 | 0.94 | 0.84 | 0.80 | 0.75 |
| HS2 | 1.16 | 1.12 | 1.26 | 1.20 | 1.20 | 1.42 | 0.98 | 1.04 | 0.69 | 0.80 | 0.76 | 0.58 |
| HS3 | 1.14 | 1.13 | 1.20 | 1.23 | 1.25 | 1.38 | 1.08 | 1.05 | 0.90 | 0.90 | 0.81 | 0.73 |
| HS4 | 1.19 | 1.14 | 1.23 | 1.28 | 1.25 | 1.42 | 1.20 | 1.15 | 0.93 | 0.92 | 0.82 | 0.74 |
| SD | 0.02 | 0.01 | 0.04 | 0.04 | 0.03 | 0.05 | 0.17 | 0.05 | 0.12 | 0.06 | 0.03 | 0.08 |

Data are expressed as the Ct ratio (of target gene to the housekeeping gene B2M), where lower values indicate *higher* amounts of target cDNA, for each of four individual seals.

both up-regulated; expression of *Itk* was significantly increased for PBMC exposed to BaP ($p < 0.01$) and CB-169 ($p = 0.01$). Likewise, *Fyn* expression was significantly increased in CB-169 samples ($p = 0.03$); the increase was marginally nonsignificant for BaP exposures ($p = 0.08$). mRNAs for IL-2 were decreased relative to controls by BaP ($p = 0.01$) and CB-169 ($p = 0.02$). Likewise, IL-1 transcription was depressed by both treatments as well (BaP $p < 0.01$, CB-169 $p = 0.03$).

### Discussion

Experimental studies using laboratory rodents and human cell lines have elucidated PAH- and HAH-sensitive endpoints of immune dysfunction of both humoral and cell-mediated immunity, and have provided evidence for various mechanisms of action in these mammalian systems (reviewed in Kerkvliet and Burleson 1994, White et al. 1994, Davila et al. 1995). Most studies have focused on the prototypic

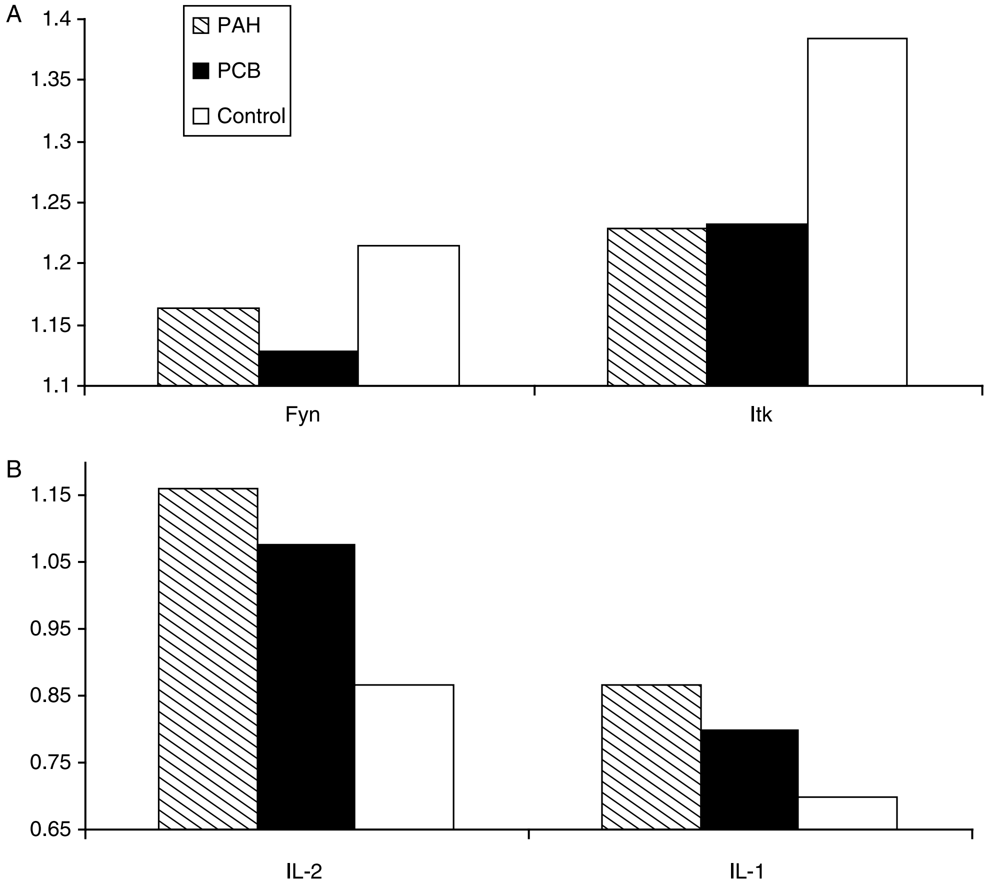

Figure 1. Gene expression of protein tyrosine kinases *Fyn* and *Itk* (A) and cytokines IL-2 and IL-1 (B) in Con A-stimulated harbor seal PBMC exposed *in vitro* to PAH (20 μM benzo[*a*]pyrene), PCB (20 μM hexachlorobiphenyl 169), and vehicle control (20 μM DMSO), based on real-time PCR. Data are mean Ct ratios ($n = 4$ seals), where lower values indicate *higher* amounts of target cDNA. Expression of the protein kinases *Fyn* and *Itk* was significantly increased following PAH and PCB exposures, whereas that of IL-2 and IL-1 was decreased.

immunotoxic PAHs BaP and 7,12-dimethylbenz[*a*]anthracene (DMBA) and their metabolites, and the prototypic immunotoxic HAH, 2,3,7,8-tetrachlorodibenzo-*p*-dioxin (TCDD). The following proposed mechanisms whereby PAHs mediate their immunosuppressive actions are not mutually exclusive, and include interaction with the intracellular aromatic hydrocarbon (Ah) receptor; disruption of lymphocyte signaling; altered interleukin production; disruption of intracellular calcium mobilization; and metabolic activation to reactive metabolites. Many of the effects of TCDD and structurally related HAHs appear to be mediated via binding to the Ah receptor; toxicity may be caused by the products of as-yet-unidentified genes induced, along with the CYP1A1 enzyme, following Ah receptor activation. However, in vivo, ex vivo, and in vitro studies of TCDD immunotoxicity have produced conflicting data with respect to target cell types and specific immune responses to exposure; these discrepancies may relate to in vitro culture conditions, genetic differences among experimental species or strains at the Ah locus, and direct vs. indirect (e.g. via effects on the endocrine system) effects on lymphoid tissues.

This study is, to our knowledge, the first to investigate effects of in vitro PAH or PCB exposure on PTK or cytokine targets in a marine mammal. Previous research on rodent and human cells, however, does provide a basis for comparison. Similarly to our findings, exposure to DMBA in a human T cell line resulted in the activation of *Fyn* and *Lck* kinases, as well as increased tyrosine phosphorylation of PLCγ1, increased formation of $IP_3$, and a sustained rise in intracellular calcium; these authors speculated that xenobiotic-induced phosphorylation mimicked antigen-receptor activation in T cells and could lead to alterations in antigen responsiveness *in vivo* (Archuleta et al. 1993). In another study from the same research group, the PTKs *Lyn* and *Syk* were activated in a human B cell line exposed to BaP metabolites, and this effect also was associated with increased intracellular calcium (Mounho and Burchiel 1998). Stimulation of tyrosine phosphorylation also was reported for murine B lymphocytes exposed to TCDD (Kramer et al. 1987, Clark et al. 1991).

Decreased production of IL-2 following PAH and PCB exposures also has been reported previously. IL-2 activity of rat splenocytes was significantly suppressed following in vitro exposure to a PCB mixture (Exon et al. 1985). Steppan et al. (1993) observed a profound suppression of IL-2 and INF-γ production in CB-169-treated mice. Similarly for PAHs, splenocytes isolated from mice exposed in vivo to DMBA or BaP showed suppressed IL-2 production (House et al. 1987, Lyte et al. 1987). In vitro exposure of mouse splenocytes to DMBA or BaP also resulted in suppression of IL-2 production (House et al. 1987, Thurmond et al. 1988, Pallardy et al. 1989).

PTKs and IL-2 apparently have not been simultaneously investigated in previous studies of PAH or HAH immunotoxicity. So far as PAH- and PCB-induced increases in *Fyn* and *Itk* transcription corresponded to increased synthesis of these proteins—thus presumably stimulating TCR signaling—depression of IL-2 gene transcription indicated incomplete TCR signal transduction. The mechanistic link between the observed phenomena is not clear and the many intervening biochemical events suggest various scenarios; potential intermediate targets (direct or indirect) include regulatory kinases and/or phosphastases, elements of the ras/MAP kinase cascade and the $Ca^{2+}$-dependent DAG/PKC and $IP_3$/calcineurin pathways, and transcription factors such as NF-AT (nuclear factor of activation in T cells), which binds to the promoter region of the IL-2 gene and is necessary to activate its transcription (Archuleta et al. 1993, Qian and Weiss 1997, Chakravarti et al. 1998, Yu et al. 2002).

The strong suppression of IL-1 expression suggested a direct inhibitory effect of the model compounds on the macrophage. Alternatively, effects of BaP and CB-169 on macrophages could have been indirect, i.e. via effects on $T_H1$ cells resulting in suppressed expression of INF-γ (a factor of macrophage activation), which in turn could reduce macrophage production of IL-1 (Steppan et al. 1993). The apparent effect on macrophage function suggests that exposure to these compounds could compromise antigen presentation/processing and macrophage support of T cell function in humoral immunity, as has been shown in BaP exposures using a mouse model (Myers et al. 1988). The ability of the model PAH and PCB to decrease production of inflammatory chemoattractive mediators such as IL-1 would imply that exposure could result in decreased host resistance to infection.

Here we have begun to explore mechanisms underlying in vitro immunomodulatory effects of PAHs and PCBs in harbor seal PBMC, focusing on alterations in gene transcription of PTK, which play a critical role in T cell activation, and IL-2, a cytokine of central importance in initiating adaptive immune responses. Our findings are consistent with those of previous researchers working with human and rodent systems and support a hypothesis of contaminant-altered lymphocyte function mediated (at least in part) by disruption of TCR signaling and cytokine production. The potential for contaminant-induced immune alterations has particularly important implications for marine mammals experiencing pathogen exposure concomitant with chronic, multiple exposures to relatively high levels of persistent organic pollutants (POPs) such as certain of the PAHs and PCBs. Reported levels of POPs in free-ranging harbor seals inhabiting many polluted areas of Europe and North America are sufficiently high to suggest that many populations may be at risk of immunotoxicity, which could manifest as diminished host resistance and increased incidence and severity of infectious disease (Reijnders 1994, Ross et al. 1996, Neale 2004, Ross et al. 2004, Neale et al. 2005). Continued investigation of the cellular and molecular pathways of PCB- and PAH-induced immune dysfunction in marine mammals will enhance our understanding of individual and population health and promote marine mammal conservation.

### Acknowledgements

We are greatly indebted to JT Harvey and crew (Moss Landing Marine Laboratories) for seal captures. This project was supported by the California Department of Fish and Game's Oil Spill Response Trust Fund through the Oiled Wildlife Care Network at the Wildlife Health Center, School of Veterinary Medicine, University of California, Davis.

### References

Abbas AK, Lichtman AH. 2003. Cellular and molecular immunology. 5th ed. Philadelphia: Saunders (Elsevier), The Curtis Center. pp 163–188, 243–274.

Aguilar A, Borrell A. 1994. Abnormally high polychlorinated biphenyl levels in striped dolphins (*Stenella coeruleoalba*) affected by the 1990–92 Mediterranean epizootic. Sci Total Environ 154:237–247.

Archuleta MM, Schieven GL, Ledbetter JA, Deanin GG, Burchiel SW. 1993. 7,12-dimethylbenz[*a*]anthracene activates protein-tyrosine kinases *Fyn* and *Lck* in the HPB-ALL human T cell line and increases tyrosine phosphorylation of phospholipase C-γ1, formation of inositol 1,4,5-triphosphate, and mobilization of intracellular calcium. Proc Natl Acad Sci 90:6105–6109.

Beckmen KB, Blake JE, Ylitalo GM, Stott JL, O'Hara TM. 2003. Organochlorine contaminant exposure and associations with hematological and humoral immune functional assays with dam age as a factor in free-ranging northern fur seal pups (*Callorhinus ursinus*). Mar Pollut Bull 46:594–606.

Brouwer A, Reijnders PJH, Koeman JH. 1989. Polychlorinated biphenyl (PCB)-contaminated fish induced vitamin A and thyroid hormone deficiency in the common seal (*Phoca vitulina*). Aquat Toxicol 15:99–106.

Chakravarti D, Calalieri EL, Rogan EG. 1998. Linear amplification mapping of polycyclic aromatic hydrocarbon-reactive sequences in the H-ras gene. DNA Cell Biol 17:529–539.

Clark GC, Blank JA, Germolec DR, Luster MI. 1991. 2,3,7,8-tetrachlorodibenzo-*p*-dioxin stimulation of tyrosine phosphorylation in B lymphocytes: Potential role in immunosuppression. Mol Pharmacol 39:495–501.

Davila DR, Davis DP, Campbell K, Cambier JC, Zigmond LA, Burchiel SW. 1995. Role of alterations in $Ca^{2+}$-associated signaling pathways in the immunotoxicity of polycyclic aromatic hydrocarbons. J Toxicol Environ Health 45:101–126.

De Swart RL, Ross PS, Vos JG, Osterhaus ADME. 1996. Impaired immunity in harbour seals (*Phoca vitulina*) fed environmentally contaminated herring. Vet Q 18(suppl. 3):S127–S128.

Exon JH, Talcott PA, Koller LD. 1985. Effect of lead, polychlorinated biphenyls, and cyclophosphamide on rat natural killer cells, Interleukin 2, and antibody synthesis. Fund Appl Toxicol 5:158–164.

Hall AJ, Law RJ, Harwood J, Ross M, Kennedy S, Allchin CR, Campbell LA, Pomeroy PP. 1992. Organochlorine levels in common seals (*Phoca vitulina*) which were victims and survivors of the 1988 phocine distemper epizootic. Sci Total Environ 115:145–162.

House RV, Lauer LD, Murray MJ, Dean JH. 1987. Suppression of T-helper cell function in mice following exposure to the carcinogen 7,12-dimethylbenz[*a*]anthracene and its restoration by Interleukin-2. Int J Immunopharmacol 9:89–97.

House RV, Pallardy MJ, Dean JH. 1989. Suppression of murine cytotoxic T-lymphocyte induction following exposure to 7,12-dimethylbenz[*a*]anthracene: Dysfunction of antigen recognition. Int J Immunopharmacol 11:207–215.

Janeway CA, Travers P, Walport M, Capra JD. 1999. Immunobiology: The immune system in health and disease. 4th ed. New York: Garland Publishing. pp 163–193, 263–305.

Jenssen BM, Haugen O, Sormo EG, Skaare JU. 2003. Negative relationship between PCBs and plasma retinol in low-contaminated free-ranging gray seal pups (*Halichoerus grypus*). Environ Res 93:79–87.

Kerkvliet NI, Brauner JA. 1987. Mechanisms of 1,2,3,4,6,7,8-heptachlorodibenzo-*p*-dioxin (HpCDD)-induced humoral immune suppression: Evidence of primary defect in T cell regulation. Toxicol Appl Pharmacol 87:18–31.

Kerkvliet NI, Burleson GR. 1994. Immunotoxicity of TCDD and related halogenated aromatic hydrocarbons. In: Dean JH, Luster MI, Munson AE, Kimber I, editors. Immunotoxicology and immunopharmacology. 2nd ed. New York: Raven Press. pp 97–121.

Kramer CM, Johnson KW, Dooley RK, Holsapple MP. 1987. 2,3,7,8-Tetrachlorodibenzo-*p*-dioxin (TCDD) enhances antibody production and protein kinase activity in murine B cells. Biochem Biophys Res Commun 145:25–32.

Kung C, Thomas ML. 1997. Recent advances in lymphocyte signaling and regulation. Front Biosci 2:207–221.

Lahvis GP, Wells RS, Kuehl DW, Stewart JL, Rhinehart HL, Via CS. 1995. Decreased lymphocyte responses in free-ranging Bottlenose dolphins (*Tursiops truncatus*) are associated with increased concentrations of PCBs and DDT in peripheral blood. Environ Health Perspect 103(Suppl. 4):67–72.

Lyte M, Blanton RH, Myers MJ, Bick PH. 1987. Effect of *in vivo* administration of the carcinogen benzo(*a*)pyrene on interleukin-2 and interleukin-3 production. Int J Immunopharmacol 9:307–312.

Martineau D, De Guise S, Fournier M, Shugart L, Girard C, Lagace A, Beland P. 1994. Pathology and toxicology of beluga whales from the St. Lawrence Estuary, Quebec, Canada. Past, present, and future. Sci Total Environ 154:201–215.

Mounho BJ, Burchiel SW. 1998. Alterations in human B cell calcium homeostasis by polycyclic aromatic hydrocarbons: Possible associations with cytochrome P450 metabolism and increased protein tyrosine phosphorylation. Toxicol Appl Pharmacol 149:80–89.

Myers MJ, Schook LB, Bick PH. 1987. Mechanisms of benzo(*a*)pyrene-induced modulation of antigen presentation. J Pharmacol Exp Ther 242:399–404.

Myers MJ, Blanton RH, Bick PH. 1988. Inhibition of IL-2 responsiveness following exposure to benzo(*a*)pyrene is due to alterations in accessory cell function. Int J Immunopharmacol 10:177–186.

Neale JCC. 2004. Persistent organic contaminants and contaminant-induced immune and health alterations in the harbor seal, *Phoca vitulina* [dissertation] Davis (CA): University of California. pp 13–60.

Neale JCC, Van de Water JA, Harvey JT, Tjeerdema RS, Gershwin ME. 2002. Proliferative responses of harbor seal (*Phoca vitulina*) T lymphocytes to model marine pollutants. Dev Immunol 9:215–221.

Neale JCC, Kenny TP, Gershwin ME. 2004. Cloning and sequencing of protein kinase cDNA from harbor seal (*Phoca vitulina*) lymphocytes. Clin Dev Immunol 11:157–163.

Neale JCC, Gulland FMD, Schmelzer KR, Harvey JT, Berg EA, Allen SG, Greig DJ, Grigg EK, Tjeerdema RS. 2005.

Contaminant loads and hematological correlates in the harbor seal (*Phoca vitulina*) of San Francisco Bay, California. J Toxicol Environ Health Part A 68:617–633.

Osterhaus ADME, Vedder EJ. 1989. No simplification in the etiology of recent seal deaths. Ambio 18:297–298.

Pallardy MJ, House RV, Dean JH. 1989. Molecular mechanism of 7,12-dimethylbenz[*a*]anthracene-induced immunosuppression: Evidence for action via the interleukin-2 pathway. Mol Pharmacol 36:128–133.

Qian D, Weiss A. 1997. T cell antigen receptor signal transduction. Curr Opin Cell Biol 9:205–212.

Reijnders PJH. 1986. Reproductive failure in common seals feeding on fish from polluted coastal waters. Nature 324:456–457.

Reijnders PJH. 1994. Toxicokinetics of chlorobiphenyls and associated physiological responses in marine mammals, with particular reference to their potential for ecotoxicological risk assessment. Sci Total Environ 154:229–236.

Ross P, De Swart R, Addison R, Van Loveren H, Vos J, Osterhaus A. 1996. Contaminant-induced immunotoxicity in harbour seals: Wildlife at risk? Toxicology 112:157–169.

Ross PS, Jeffries SJ, Yunker MB, Addison RF, Ikonomou MG, Calambokidis JC. 2004. Harbor seals (*Phoca vitulina*) in British Columbia, Canada, and Washington State, USA, reveal a combination of local and global polychlorinated biphenyl, dioxin, and furan signals. Environ Toxicol Chem 23:157–165.

Shoda LKM, Brown WC, Rice-Ficht AC. 1998. Sequence and characterization of phocine interleukin 2. J Wildl Dis 34:81–90.

Steppan L, DeKrey GK, Fowles JR, Kerkvliet NI. 1993. Polychlorinated biphenyl (PCB) induced alterations in the cytokine profile in the peritoneal cavity of mice during the course of P815 tumor rejection. J Immunol 150:134A.

Tanabe S, Iwata H, Tatsukawa R. 1994. Global contamination by persistent organochlorines and their ecotoxicological impact on marine mammals. Sci Total Environ 154:163–177.

Thurmond LM, House RV, Lauer LD, Dean JH. 1988. Suppression of splenic lymphocyte function by 7,12-dimethylbenz[*a*]anthracene (DMBA) *in vitro*. Toxicol Appl Pharmacol 93:369–377.

Van Loveren H, Ross PS, Osterhaus ADME, Vos JG. 2000. Contaminant-induced immunosuppression and mass mortalities among harbor seals. Toxicol Lett 112–113:319–324.

Vos JG, Luster MI. 1989. Immune alterations. In: Kimbrough RD, Jensen S, editors. Halogenated biphenyls, terphenyls, naphthalenes, dibenzodioxins and related products. Amsterdam: Elsevier Science Publishers. pp 295–322.

Weiss A, Littman DR. 1994. Signal transduction by lymphocyte antigen receptors. Cell 76:263–274.

White KL, Jr. 1986. An overview of immunotoxicology and carcinogenic polycyclic aromatic hydrocarbons. J Environ Sci Health C4:163–202.

White KL Jr, Kawabata TT, Ladics GS. 1994. Mechanisms of polycyclic aromatic hydrocarbon immunotoxicity. In: Dean JH, Luster MI, Munson AE, Kimber I, editors. Immunotoxicology and immunopharmacology. 2nd ed. New York: Raven Press. pp 123–142.

Yu D, Kazanietz MG, Harvey RG, Penning TM. 2002. Polycyclic aromatic hydrocarbon o-quinones inhibit the activity of the catalytic fragment of protein kinase C. Biochemistry 41:11888–11894.

